# When fungi knock, nitrilases unlock the auxin arsenal

**DOI:** 10.1093/plcell/koag295

**Published:** 2026-09-23

**Authors:** Jiajun Wang, Yueyao Wang

**Affiliations:** Assistant Features Editor, the Plant Cell, American Society of Plant Biologists; School of Life Sciences, Xiamen Key Laboratory of Plant Genetics, Xiamen University, Xiamen 361102, China; School of Advanced Agricultural Sciences, Peking University, Beijing 100871, China

*Botrytis cinerea*, the causal agent of gray mold, is a devastating necrotrophic pathogen that infects more than 1,400 plant species and causes substantial agricultural losses globally (Elad et al. 2015). Plants perceive fungal chitin through cell surface–localized pattern-recognition receptors such as CHITIN ELICITOR RECEPTOR KINASE 1 (CERK1) to initiate pattern-triggered immunity (PTI) (Yang et al. 2022). While plant hormones, such as auxin, are known to bolster defense responses against *B. cinerea* (Llorente et al. 2008), a fundamental question has remained: how do plants rapidly activate auxin biosynthesis upon pathogen attack, and what molecular mechanism links chitin-triggered CERK1 signaling to the auxin pathway? Reporting in new work, **Jiarui Zheng and colleagues** (Zheng et al. 2026) revealed that CERK1 directly phosphorylates nitrilases to promote indole-3-acetonitrile (IAN)-dependent auxin biosynthesis, thereby strengthening host resistance against gray mold.

To investigate the downstream signaling cascade of chitin-induced PTI, the authors performed LC-MS/MS profiling to identify Arabidopsis CERK1-interacting proteins following chitin treatment. Through a series of protein–protein interaction assays, they confirmed that CERK1 directly binds nitrilase 1 (NIT1), NIT2, and NIT3, enzymes known to redundantly convert IAN into indole-3-acetic acid (IAA). Nitrilase activity assays confirmed that NIT1, NIT2, and NIT3 were all capable of catalyzing the conversion, with NIT2 exhibiting the highest activity. Mutation of cysteine 186, which is located in the IAN-binding pocket, abolished the ability of NIT3 to convert IAN into IAA. Moreover, the *nit1 nit2 nit3* (*ntm*) triple mutant completely lost the ability of both IAN-to-IAA conversion and activation of auxin signaling in response to exogenous IAN.

To determine whether NIT nitrilases function downstream of CERK1, the authors analyzed the disease resistance phenotypes of the *ntm* triple mutant following *B. cinerea* infection and assessed the contribution of NIT-mediated IAN-to-IAA conversion to pathogen resistance. Similar to the *cerk1* mutant, the *ntm* triple mutant was significantly more susceptible to *B. cinerea*. In contrast, overexpression of any of the 3 *NIT genes* (*NIT1*, *NIT2*, and *NIT3*) boosted resistance, and individual *NIT* expression successfully rescued *ntm* susceptibility. Exogenous application of IAN increased resistance to *B. cinerea* in wild-type plants but failed to rescue the susceptibility of the *ntm* mutant, whereas exogenous IAA treatment significantly enhanced resistance in both wild-type and *ntm* plants. These results indicate that NIT-mediated IAN-to-IAA conversion plays an important role in plant resistance to *B. cinerea*. Consistently, the catalytically inactive NIT3 mutant (C186A) failed to complement the susceptible phenotype of the *ntm* mutant, confirming that NIT-driven disease resistance strictly requires catalytic IAN-to-IAA conversion.

Mechanistically, the authors found that chitin treatment strengthens the interactions between CERK1 and NITs and drives CERK1-mediated phosphorylation of NIT2 in planta. In vitro kinase assays mapped this phosphorylation predominantly to threonine 149 (T149) and serine 150 (S150) on NIT2. Non-phosphorylatable mutations (T149A/S150A) rendered NIT2 unable to complement the *ntm* mutant upon infection. Molecular docking and computational analyses further revealed that T149 and S150 are positioned at the edge of the predicted IAN-binding pocket, and that their phosphorylation enhances the IAN-binding affinity of NIT2. Accordingly, CERK1 enhanced the catalytic activity of NIT2 in converting IAN to IAA, and this activation was potentiated by chitin perception.

Interestingly, although the *ntm* triple mutant exhibited no obvious defects in growth or altered auxin levels under normal conditions, auxin accumulation induced by *B. cinerea* infection is severely impaired. These results suggest that NIT1/2/3 are dispensable for developmental auxin homeostasis but essential for pathogen-induced auxin spikes, revealing a context-dependent role of the NIT-mediated IAN pathway in plant immunity.

Taken together, these findings demonstrate that during *B. cinerea* infection, chitin triggers CERK1-mediated phosphorylation of NIT1/2/3 nitrilases, promoting their catalytic conversion of IAN to IAA to boost plant resistance (Fig. 1). Importantly, this mechanism is conserved in tomato, providing potential molecular targets for the genetic improvement of gray mold resistance in crops. More broadly, this study reveals that the abundant endogenous IAN pool serves as a readily accessible “auxin precursor reservoir” for rapid IAA biosynthesis upon pathogen attack, offering new insights into how plants dynamically repurpose metabolic reserves to balance growth and defense.

**Figure 1:**
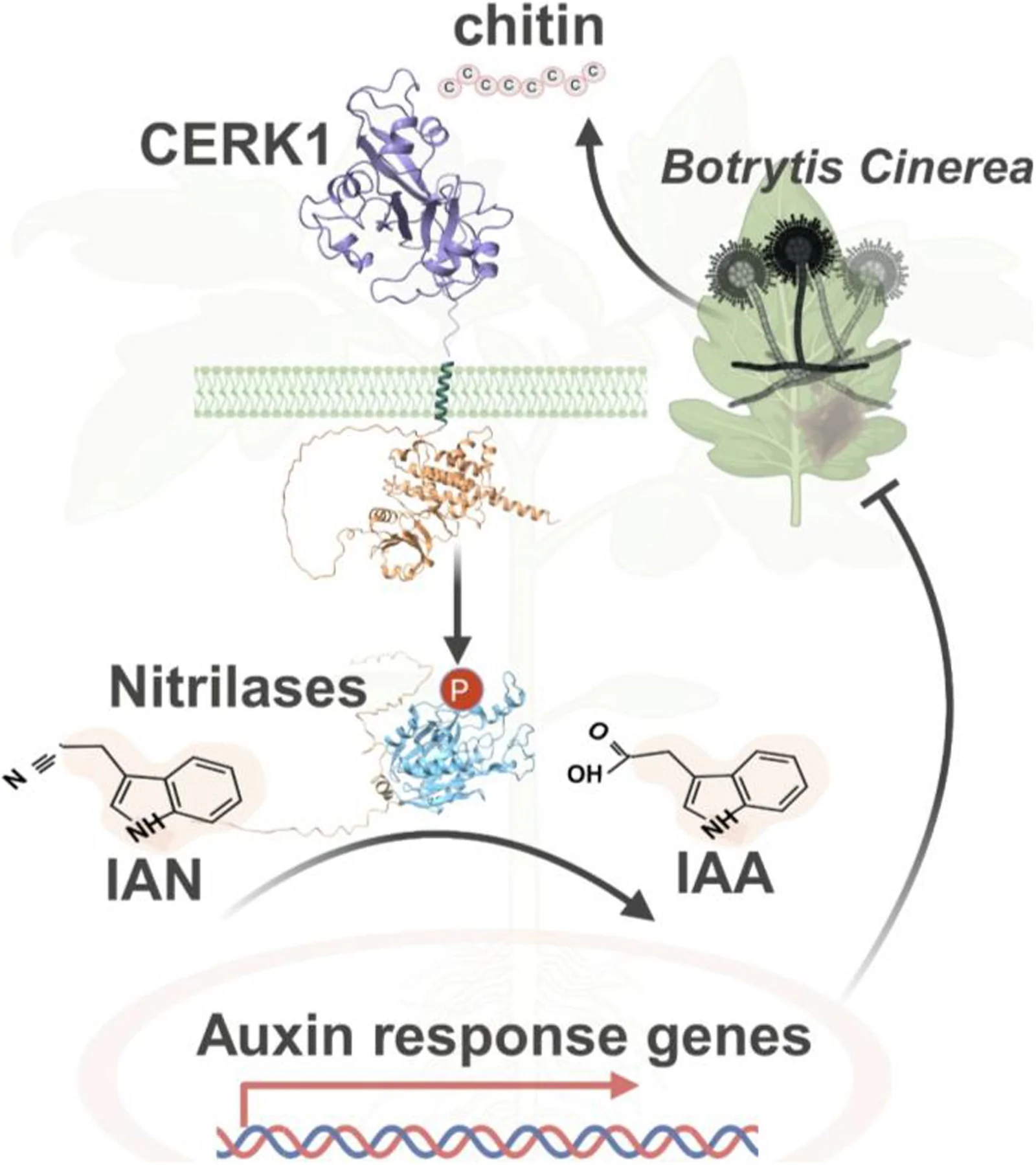
Schematic model of CERK1-mediated phosphorylation of nitrilases in plant defense against *Botrytis cinerea*. Chitin, a component of the *B. cinerea* cell wall, is recognized by the plant pattern-recognition receptor CERK1. CERK1 then phosphorylates nitrilases, enhancing their ability to bind IAN and catalyze its conversion to IAA. The resulting increase in IAA promotes the expression of auxin-responsive genes, thereby enhancing plant resistance to *B. cinerea*. Reprinted from Zheng et al. (2026), Figure 7D.

## Recent related articles in *The Plant Cell*

- Zhang et al. (2025) revealed that a MYC2–LBD40/42–CRL3^BPM4^ module that fine-tunes *Botrytis cinerea* defense in tomato by balancing immune activation and plant growth.
- Sun et al. (2025) revealed a CPK5–MORC1–NPR1–TGA module that transduces Ca^2+^ signals to activate defense gene expression and promote broad-spectrum disease resistance in Arabidopsis.

## Data Availability

N/A.

## References

[koag295-B1] Elad Y, Vivier M, Fillinger S. 2015. *Botrytis*, the good, the bad and the ugly. In: Fillinger S, Elad Y, editors. Botrytis—the fungus, the pathogen and its management in agricultural systems. Springer. p. 1–15.

[koag295-B2] Llorente F et al 2008. Repression of the auxin response pathway increases Arabidopsis susceptibility to necrotrophic fungi. Mol Plant. 1:496–509. 10.1093/mp/ssn025. 19825556

[koag295-B3] Sun C et al 2025. The kinase CPK5 phosphorylates MICRORCHIDIA1 to promote broad-spectrum disease resistance. Plant Cell. 37:koaf051. 10.1093/plcell/koaf051. PMC11952926 40085777

[koag295-B4] Yang C, Wang E, Liu J. 2022. CERK1, more than a co-receptor in plant-microbe interactions. New Phytol. 234:1606–1613. 10.1111/nph.18074. 35297054

[koag295-B5] Zhang J et al 2025. Fine-tuning of MYC2-mediated Botrytis defense response by the LBD40/42-CRL3BPM4 module in tomato. Plant Cell. 37:koaf258. 10.1093/plcell/koaf258. 41134692

[koag295-B6] Zheng J et al 2026. CERK1-mediated phosphorylation of nitrilases activates auxin biosynthesis and promotes resistance to Botrytis cinerea. Plant Cell. 10.1093/plcell/koag233. 42607133

